# Apoptosis Induced by 13-S-hydroxyoctadecadienoic acid in the Breast Cancer Cell Lines, MCF-7 and MDA-MB-231

**Published:** 2013-04

**Authors:** Masoumeh Tavakoli Yaraki, Fatemeh Karami Tehrani

**Affiliations:** 1 Department of Clinical Biochemistry, Cancer Research Laboratory, Faculty of Medical Sciences, Tarbiat Modares University, Tehran, Iran

**Keywords:** Apoptosis, MCF-7, MDA-MB-231, PPAR-δ, 13-S-hydroxyoctadecadienoic acid

## Abstract

***Objective(s)*** *: *The 15-Lipoxygenase-1(15-LOX-1) pathway has become of considerable interest as a promising molecular approach for the modulation of cancer cell growth. 13-S-hydroxyoctadecadienoic acid (13(S)-HODE) is a main metabolite of 15-LOX-1 which is proposed to influence the cancer cell’s growth. This study aims to investigate the role of 13(S)-HODE in the regulation of cell growth and apoptosis in the breast cancer cell lines.

***Materials and Methods*** *:* MTT assay was used to examine the cytotoxic effect of 13(S)-HODE in the breast cancer cells, MCF-7 and MDA-MB-231.Annexin-V-FITC staining and cell cycle analysis were performed using flow cytometry. The effect of 13(S)-HODE on the expression level of Peroxisome proliferator-activated receptors-δ (PPAR-δ) was also evaluated.

***Results*** *:* The results demonstrated that 13(S)-HODE inhibited cell growth in a dose and time dependant manner in MCF-7 and MDA-MB-231 cell lines. The reduction of cell growth was associated with the induction of cell cycle arrest and apoptosis in the breast cancer cell lines. Moreover, PPAR-δ was down-regulated in response to 13(S)-HODE administration.

***Conclusion:*** This study conducted evidences in to the stimulatory effect of 13(S)-HODE on the inhibition of cell growth and induction of apoptosis in the breast cancer cell lines.

## Introduction

Breast cancer is considered as the leading cause of cancer-related mortality among women in the world (1). Many scientific efforts have been focused on finding better therapeutic strategies and new molecular approaches to reduce the mortality.

The homeostasis of cancer cells depends on the balance between apoptosis and cell proliferation(2); therefore, identification of the critical mechanisms associated with the loss of apoptosis and increased cell proliferation is important for molecular targeting. 15-lipoxygenase-1(15-LOX-1) belongs to the non-heme iron-containing di-oxygenases family which is expressed mainly in the reticulocytes, eosinophils, macrophages, and airway epithelium(3). 15-LOX-1 catalyzes the dioxigenation of linoleic acid at the 13^th^ carbon and produces 13-S- hydroxyoctadecadienoic acid (13(S)-HODE) (4). Linoleic acid is an essential poly unsaturated fatty acid in the human diet and changes in the level of 13(S)-HODE is associated with the pathological conditions (5). It has been shown that down-regulation of 15-LOX-1 in the lung(6), colorectal (7)esophageal (8), pancreatic(9) and breast(10) cancer, associated with the reduction of 13(S)-HODE, leads to the cancer progression (5,6,9,11). Moreover, 13(S)-HODE is implicated to the cell differentiation and apoptosis in colorectal cancer (12-15) and 13(S)-HODE acts as a modulator of tumor invasion and metastasis (16,17). 

Peroxisome proliferator -activated receptors (PPARs) are ligand-activated transcription factors belong to the nuclear receptor super family (18). Three isoforms of PPARs have been recognized including PPARα, PPAR δ and PPARγ. The involvement of PPARs in lipid homeostasis and cellular differentiation, tumorigenesis and cancer development have been well-established so far (19,20). Polyunsaturated fatty acids such as 13(S)-HODE and their related metabolites modulate PPAR-δ expression and function (19). It has been shown that 13(S)-HODE significantly reduces PPAR-δ expression and activity in colorectal cancer (21). 15-LOX-1 expression is down-regulated in the breast cancer. However, the mechanistic contribution in the breast tumorigenesis remains to be elucidated(10). On the other hand, the involvement of 13(S)-HODE in the regulation of breast cancer cell growth is not clearly determined. Accordingly, the current study is designed to evaluate the effect of 13(S)-HODE on the inhibition of cell growth, induction of apoptosis and cell cycle distribution in human breast cancer cell lines, MDA-MB-231 and MCF-7. The expression level of PPAR-δ as an endogenous receptor for 13(S)-HODE has been assessed in both cell lines. The results demonstrate that 13(S)-HODE suppresses the growth of MDA-MB-231 and MCF-7 cells which is associated with the cell cycle arrest and induction of apoptosis. The effect of 13(S)-HODE is also accompanied by the reduction of PPAR- δ expression level.

## Materials and Methods

*Chemical materials and Cell culture*

RPMI 1640, trypsin/EDTA, Nacl/Pi,penicillin and streptomycin were obtained from Gibco (Rockville, USA). 13-S-hydroxyoctadecadienoic acid (13(S)-HODE) was provided by Cayman Chemicals (Michigan,USA). Annexin-V-FITC apoptosis detection kit, Propidium Iodid(PI) ,MTT [3-(4,5-dimethyltiazol-2-yl)-2,5-diphenyltetrazolium bromide], dimethylsulfoxide were purchased from Sigma-Aldrich (Munich,Germany).

Human breast cancer cell lines, MCF-7 and MB-MDA-231 were purchased from Pasture Institute of Iran. Cells were cultured in RPMI 1640 containing 10% (v/v) fetal bovine serum, 100 U/ml of penicillin and 100 µg/ml of streptomycin and were maintained at 37 ºC with an atmosphere of 5% CO_2 _and 100% humidity. Cells were used freshly and collected by tripsinization at the confluence of 70-100%. The results of 13(S)-HODE on the breast cancer cell growth were always compared to the cells treated with medium alone and cells treated with Ethanol as a solvent of 13(S)-HODE, in order to validate the effect of 13(S)-HODE. Untreated cells and cells treated with different concentrations of Ethanol equal to 13(S)-HODE had no effect on MCF-7 and MDA-MB-231 viability, apoptosis and cell cycle distribution (data not shown).

*Cell viability assay: MTT assay*

MTT assay was used to determine the viability of treated and untreated cells. MCF-7 and MB-MDA-231 cell lines were cultured in 96-well plates at 5*10^3^ cell/well in RPMI 1640 (supplemented with 10% fetal bovine serum, 100 U/ml of penicillin and 100 µg/ml of streptomycin) in 5% CO2 at 37 ºC until nearly confluent. 13(S)-HODE was dissolved in ethanol and cells were treated with the indicated concentrations of 13(S)-HODE and incubated in RPMI 1640 (supplemented with 2% FBS) for 24, 48 and 72 hr. Cells treated with the Ethanol (as a vehicle) were considered as a control. 20 µl of MTT (5mg/ml in PBS) was added to each well and cells were incubated for 4 hr at 37 ºC. The supernatant of each well was removed and formazan crystals were dissolved in 200 µl of dimethylsulfoxide, respectively. The absorbance was read by microplate reader at 570 nm ( Bio-Rad, Hercules, CA, USA). Treated groups were compared to vehicle controls and the percentages of viable cells were reported, respectively. The Data represent at least three separate repeated experiments.

*Flow cytometric analysis of apoptosis*

To quantify the apoptotic cell death, Annexin-V-FITC kit was applied and the results were analyzed using flow cytometry according to the manufacturer’s specifications. Briefly, cells were harvested at the density of 1*10^6 ^cells/ml following treatment with various concentrations of 13(S)-HODE (Cells treated with the Ethanol (as a vehicle) were considered as a control.) and washed twice with PBS. Cell pellets were resuspended in 500 µl of 1×binding buffer, following gentle vortex, 5 µl of annexin-V-FITC and 5µl of PI were added to each sample. Cells were incubated 10 min at room temperature in the dark and further analyzed by FACSCalibur flow cytometer (BectonDickinson, SanJose, USA) using the supplied software in the instrument (BD CellQuest software).

*Analysis of Peroxisome proliferator -activated receptors-δ* *(* *PPAR-δ) mRNA expression*

Trizol (Invitrogen, Grand Island, USA) was used to extract total RNA from cells according to the manufacturer’s instructions. To evaluate RNA integrity agarose gel electrophoresis was applied. Then 1 µg of total RNA was reverse-transcribed to synthesize single-stranded cDNA using High Capacity RNA-to-cDNA Kit according to the manufacturer’s specifications (Invitrogen, Grand Island, USA). Specific cDNA was used as a template and quantitative real-time RT-PCR analysis of PPAR-δ was performed using the SYBR Green kit (Qiagen) in an ABI 7500 Sequence Detection System (Applied Biosystems) according to the instructions and the relative gene expression levels were normalized to the expression level of GAPDH (housekeeping gene). The relative expression level was evaluated using 2^-ΔΔCt ^analysis method. RT-PCR analysis was also performed and the products were analyzed by agarose gel electrophoresis 2% (w/v).

The following primer sets were applied:

PPAR-δ (NM-006238): Forward, 5’- agcagcctcttcctcaacgacag -3’, and Reverse, 5’- ggtctcggttcggtcttcttgat -3’, 

glyceraldehydes-3-phosphatedehydrogenase(GAPDH) (NM-002046):

Forward: 5’- aatgaccccttcattgacctc-3’, Reverse: 5’-agttgtcatggatgaccttgg-3’

*Cell cycle analysis*

PI staining was used to measure the DNA content and cell distribution. The MCF-7 and MB-MDA-231 cells were treated with different concentrations of 13(S)-HODE for 48 hr (Cells treated with the Ethanol (as a vehicle) were considered as a control). Then cells were harvested and fixed with 70% (v/v) ice-cold ethanol and washed twice with ice-cold PBS and stained with 0.1mg/ml of RNase A (Sigma) and 0.05 mg/ml of PI (Sigma) for 1 hr at 37 ºC in the dark. 

Cell cycle distribution and the percentage of cells in various cell cycle phases were determined using FACSCalibur flow cytometer (BectonDickinson, SanJose,USA) and inbuilt software ( BD CellQuest software). The sub-G1 population was considered as apoptotic cells.

*Statistical analysis*

Non-parametric one-way analysis of variance (ANOVA) with *post hoc* Dennet’s test was conducted to analyze and compare data using software GraphPad Prism. Data was presented as mean±SD and all experiments were repeated at least three times. Differences were taken significant for *P *<0.05 and determined by asterisk in corresponding Figure.

## Results

*The effect of 13(S)-HODE on the cell growth inhibition in the breast cancer cell lines*

**Figure 1:**
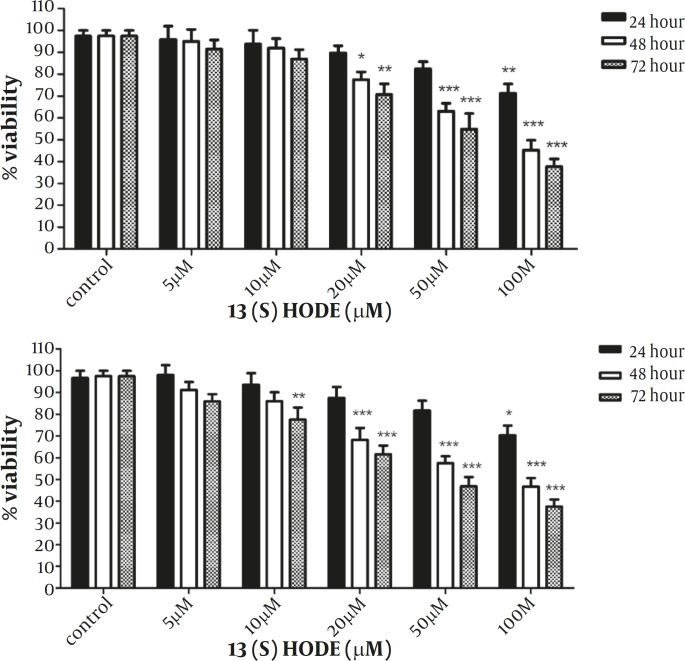
13(S)-HODE inhibited cell growth in breast cancer cell lines.

The effect of 13(S)-HODE was examined on the growth of MCF-7 and MDA-MB-231 breast cancer cell lines. Cells were treated with different concentrations of 13(S)-HODE (5-100 µM) and incubated in RPMI 1640 9 supplemented with 2% FBS) for 24, 48 and 72 hr and the cell viability was measured by MTT assay. As indicated in Figure 1A, a significant reduction of MCF-7 cell viability was observed in a time and dose dependent manner. Treatment of MCF-7 cells with 100 µM of 13(S)-HODE reduced the cell viability about 53.25% and 62.5% after 48 and 72 hr, respectively. Furthermore, treatment of MDA-MB-231 cells with 13(S)-HODE resulted in a significant decrease in the percentage of viable cells in a time and dose dependent manner. Treatment of MDA-MB-231 cells with 100 µM of 13(S)-HODE reduced the cell viability about 57.75% and 62.25% after 48 and 72 hr, respectively (Figure 1.B). The IC50 value (the effective dose of 13(S)-HODE that causes 50% growth inhibition), for MCF-7 and MDA-MB-231 after 48 hr were 76.3 μM.and 80.23 μM, respectively.

*The Effect of 13(S)-HODE on the cell cycle progression*

**Figure 2:**
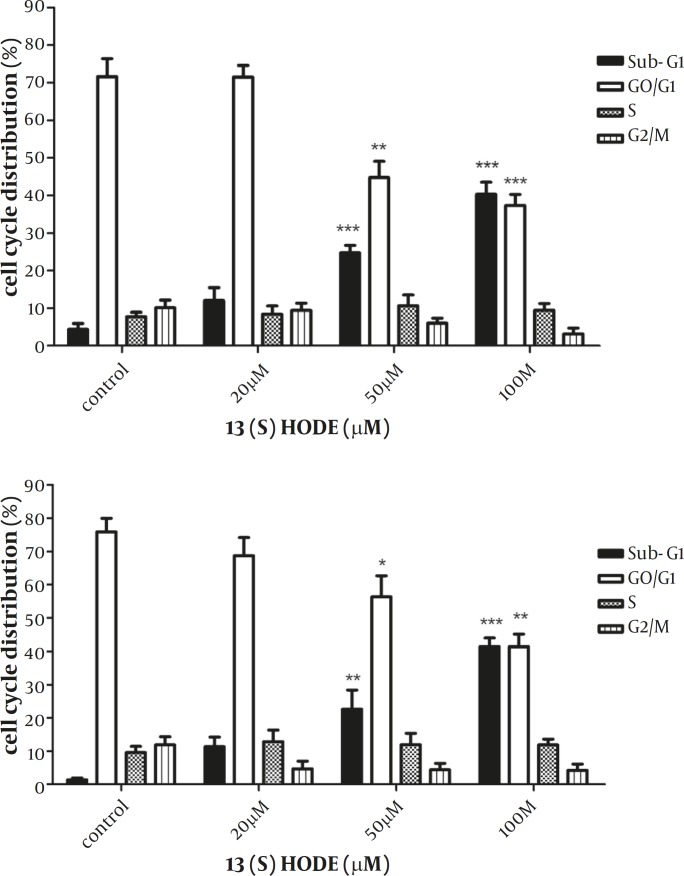
The effect of 13(S)-HODE on cell cycle distribution in breast cancer cell lines

The effect of 13(S)-HODE on the cell cycle progression was studied in the breast cancer cell lines. MCF-7 and MDA-MB-231 cells were treated with 20, 50, 100 µM of 13(S)-HODE and incubated in RPMI 1640 (supplemented with 2% FBS) for 48 hr and cell cycle analysis were performed using flow cytometry. A significant increase in the percentage of Sub-G1 population in MCF-7 (*P<*0.001) and MDA-MB-231 (*P<*0.001) was observed (Figure 2). The elevation in the sub-G1 population occurred in a dose dependant manner in both cell lines. The results demonstrated that the treatment of the cells with 13(S)-HODE were associated with the disturbance in the cell cycle progression, raising the cell cycle arrest in the breast cancer cell lines.

*The effect of 13(S)-HODE on the induction of apoptosis in MCF-7 and MDA-MB-231 cell lines*

**Figure 3:**
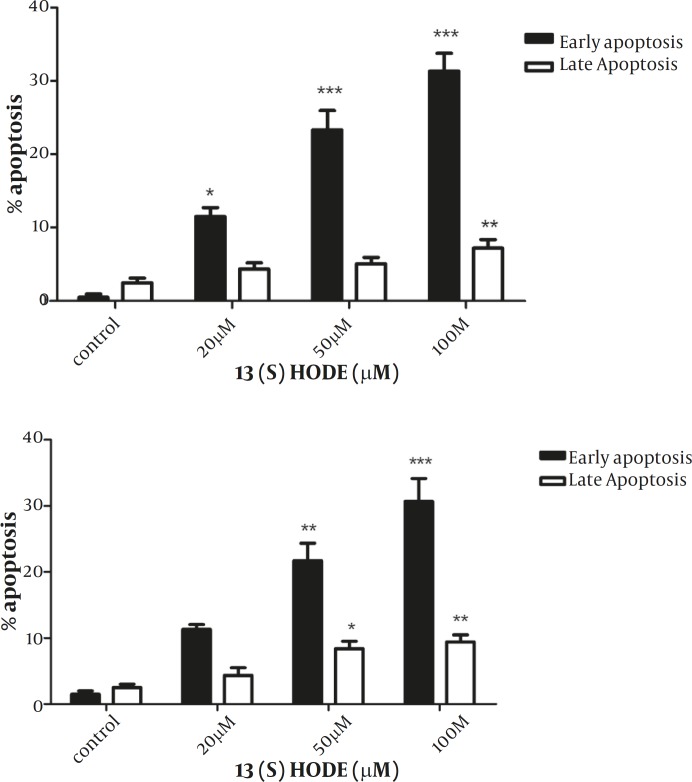
13(S)-HODE induced apoptosis in breast cancer cell lines

To examine the effect of 13(S)-HODE on the induction of apoptosis, annexin-V and PI double staining method was employed and analyzed using flow cytometry with BD CellQuest software. According to the instruction, annexin-V positive, PI negative cells are recognized as early apoptotic and annexin-V positive, PI positive are considered as late apoptotic cells. MCF-7 and MDA-MB-231 cells were treated with 20, 50 and 100 µM of 13(S)-HODE and incubated in RPMI 1640 (supplemented with 2% FBS) for 48 hr, and then analyzed by flow cytometry. Annexin-negative and PI –negative cells were considered as controls. A significant increase was observed in the percentages of early (*P<*0.001) and late (*P<*0.01) apoptotic cells for MCF-7 cells (Figure 3A). A significant elevation in the level of early (*P<*0.001) and late (*P<*0.01) apoptotic cells was also detected in MDA-MB-231 cells (Figure 3B). The treatment of the cells through increasing the concentrations of 13(S)-HODE resulted in a notable shift from viable to apoptotic cells in a dose dependant manner in both cell lines. The rate of apoptotic cells was ranged from 3.5% to 43.28% for MCF-7 and 2.99% to 40.2 % for MDA-MB-231.

*The effect of 13(S)-HODE on the PPAR-δ gene expression in the breast cancer cell lines*

**Figure 4:**
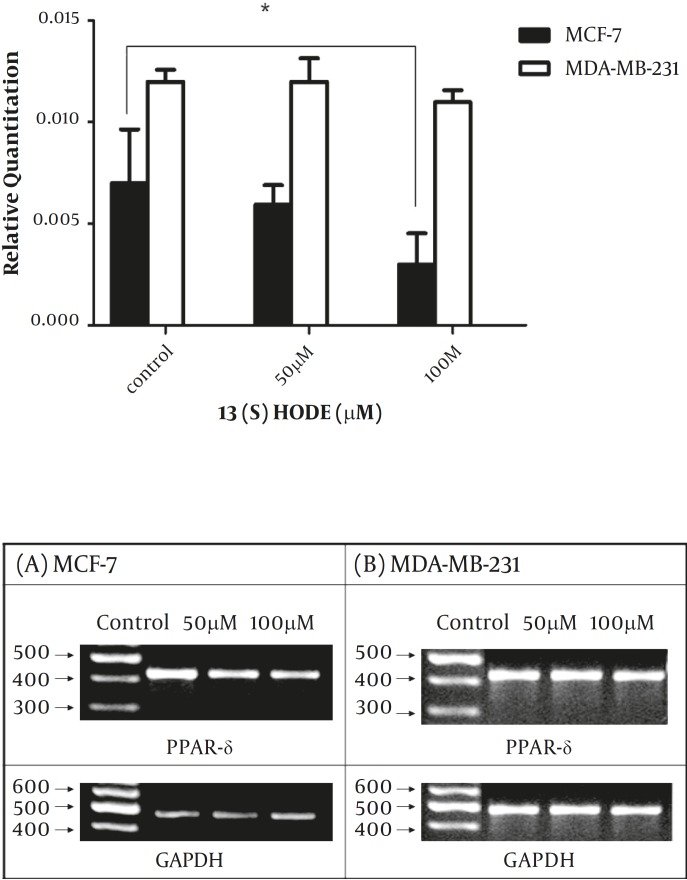
The effect of 13(S)-HODE on the expression level of PPAR-δ in breast cancer cells.

The expression level of PPAR-δ was studied following the treatment by 13(S)-HODE to determine whether PPAR-δ expression is affected by 13(S)-HODE. Cells were treated in different concentrations of 13(S)-HODE (50 and 100 µM) for 48 hr and then analyzed by real-time RT-PCR experiments. The results demonstrated that PPAR-δ expression level reduced in response to 13(S)-HODE in MCF-7 cell line, while 13(S)-HODE had no significant effect on the PPAR- δ expression level in MDA-MB-231 (Figure 4A), the result of RT-PCR analysis also supported the above data (Figure 4B).

## Discussion

Liloneic acid is an essential poly-unsaturated fatty acid which produces 13(S)-HODE via 15-lipoxygenase (15-LOX-1) pathway (22). 13(S)-HODE mediates important molecular functions in mammalian cells including cellular adhesion(16) and proliferation(12). Aberrant expression of enzymes involved in the oxygenation of fatty acids is correlated with the modulation of cancer cell-growth. The subsequent effect depends on the expression of enzyme, type of formed metabolite and type of cancer involved. It has been well-established that 15-LOX-1 mediates anti-tumorigenic effect in various cancers, however, the correlated mechanism is not unraveled yet (10,17,23-25). Recent studies have shown that 13(S)-HODE mediates inhibition of cell-growth and induction of apoptosis in malignant cells(23). The pro-apoptotic role of 13(S)-HODE has been shown in colorectal cancer (5,7). It has been revealed that the reduction of 15-LOX-1 expression and 13(S)-HODE formation are associated with the progression of breast cancer (10) These findings have emphasized on the possible effect of 13(S)-HODE on the inhibition of different cancer progression. The emergence of 13(S)-HODE as a novel molecular approach to trigger cell death pathways and lack of solid evidences on the precise role of 13(S)-HODE in the regulation of breast cancer cell growth, motivated us to study the effect of 13(S)-HODE in the regulation of cell growth and apoptosis in breast cancer which is the most leading cause of death in women. Treatment of MCF-7 and MDA-MB-231 cells with increasing concentrations of 13(S)-HODE inhibited cell growth in a dose and time-dependent manner in both cell lines. Our data is consistent with the inhibitory effect of exogenous 13(S)-HODE on the growth of colorectal (7) and Leukemia cell lines (23). The results revealed that the effect of 13(S)-HODE on the breast cancer cell growth is associated with an increase in the percentage of sub-G1 population which is consistent with the effective role of 13(S)-HODE in the regulation of cell-growth in human colon cancer cells (14) Our findings showed that the growth inhibition mediated by 13(S)-HODE was accompanied by a significant induction of apoptosis in a dose dependent manner in MCF-7 and MDA-MB-231 cell lines. In addition, it has been reported that the activation of PPAR-δ is correlated with cancer development in liver (26), prostate (27) , breast (20,28,29) cancers. Ligand activation of PPAR-δ increases breast cancer cell proliferation and promotes mammary gland cancer development (27-29); polyunsaturated fatty acids and their related metabolites modulate PPAR-δ expression and function (19); therefore, the possible association of 13(S)-HODE with PPAR-δ in the breast cancer cells has been evaluated in this study and it has been demonstrated that 13(S)-HODE significantly reduces PPAR-δ expression and activity in colorectal cancer (21). Our findings indicate that PPAR-δ is down-regulated in response to 13(S)-HODE in MCF-7, however, no significant differences have been observed in MDA-MB-231. This is in line with the previous report which indicated that the growth of estrogen receptor negative breast cancer cells were not influenced by PPAR- δ(27). Our findings have demonstrated the effective role of 13(S)-HODE on the expression of this receptor, which is in consistency with the down-regulation of PPAR beta/delta by 13(S)-HODE in human colorectal cancer(21). However, reports about the effect of PPAR-δ on the regulation of breast cancer cell growth are in conflict. It has been reported that the ligand activation of PPAR-δ inhibits mouse mammary gland cells’ growth (20), while the activation of PPAR-δ has been involved in the proliferation of mammary epithelial cells(29). This conflicting data can be related to the differences in the type of ligands, analysis procedures and involvement of other molecular pathways. However, it might be postulated that PPAR-δ may be involved in the apoptosis induced by 13(S)-HODE in breast cancer.

To characterize whether the 13(S)-HODE mediated growth inhibition is dependent on the estrogen activity, estrogen receptor positive (MCF-7) and negative (MDA-MB-231) breast cancer cell lines are used in this study. Our results demonstrate that no significant differences are observed in the sensitivity of the cells to the effect of 13(S)-HODE. Reports on the stimulatory or inhibitory effect of 13(S)-HODE on the cancer cell growth are discussed controversially. It has been reported that 13(S)-HODE in combination with EGF (epidermal growth factor) exhibit mitogenic effect on the SHE (Syrian hamster embryo) (30) rat hepatoma cells(31). Additionally, the elevation of 15-LOX-1 expression level and 13(S)-HODE formation have been reported in the tumors of prostate (32). Exogenous 13(S)-HODE has also increased the level of cell proliferation in prostate cancer cell lines (33). The results of other studies as well as our data support the anti-tumorigenic effect of 13(S)-HODE in cancer cells(23). This conflicting data on the significant role of 13(S)-HODE in the regulation of cell-growth can be explained by the tissue specificity of 13(S)-HODE and other possible molecular mechanisms that might be involved in the 15-LOX-1 signaling pathway. It is clear that further studies are required to characterize the precise molecular mechanism by which 13(S)-HODE affects cancer cell-growth.

## Conclusion

Taken together, the data presented here demonstrates that 13(S)-HODE induces apoptosis and cell cycle arrest in estrogen receptor positive and negative breast cancer cell lines. The result of present study underlines the mediatory role of 13(S)-HODE for the induction of apoptosis in breast cancer cells and provides evidence on the possible relevance of PPAR-δ in the regulation of cell growth mediated by 13(S)-HODE; however, further studies are needed to clarify the specific role of PPAR family. Based on the data presented here and other investigations, 13(S)-HODE might emerge as a promising molecular approach for the induction of apoptosis in breast cancer. 
